# Lipid Lowering and HDL Raising Gene Transfer Increase Endothelial Progenitor Cells, Enhance Myocardial Vascularity, and Improve Diastolic Function

**DOI:** 10.1371/journal.pone.0046849

**Published:** 2012-10-04

**Authors:** Stephanie C. Gordts, Eline Van Craeyveld, Ilayaraja Muthuramu, Neha Singh, Frank Jacobs, Bart De Geest

**Affiliations:** Center for Molecular and Vascular Biology, Catholic University of Leuven, Leuven, Belgium; Institute of Clinical Medicine, National Cheng Kung University, Taiwan

## Abstract

**Background:**

Hypercholesterolemia and low high density lipoprotein (HDL) cholesterol contribute to coronary heart disease but little is known about their direct effects on myocardial function. Low HDL and raised non-HDL cholesterol levels carried increased risk for heart failure development in the Framingham study, independent of any association with myocardial infarction. The objective of this study was to test the hypothesis that increased endothelial progenitor cell (EPC) number and function after lipid lowering or HDL raising gene transfer in C57BL/6 low density lipoprotein receptor deficient (LDLr^−/−^) mice may be associated with an enhanced relative vascularity in the myocardium and an improved cardiac function.

**Methodology/principal findings:**

Lipid lowering and HDL raising gene transfer were performed using the E1E3E4-deleted LDLr expressing adenoviral vector AdLDLr and the human apolipoprotein A-I expressing vector AdA-I, respectively. AdLDLr transfer in C57BL/6 LDLr^−/−^ mice resulted in a 2.0-fold (p<0.05) increase of the circulating number of EPCs and in an improvement of EPC function as assessed by *ex vivo* EPC migration and EPC adhesion. Capillary density and relative vascularity in the myocardium were 28% (p<0.01) and 22% (p<0.05) higher, respectively, in AdLDLr mice compared to control mice. The peak rate of isovolumetric relaxation was increased by 12% (p<0.05) and the time constant of isovolumetric relaxation was decreased by 14% (p<0.05) after AdLDLr transfer. Similarly, HDL raising gene transfer increased EPC number and function and raised both capillary density and relative vascularity in the myocardium by 24% (p<0.05). The peak rate of isovolumetric relaxation was increased by 16% (p<0.05) in AdA-I mice compared to control mice.

**Conclusions/Significance:**

Both lipid lowering and HDL raising gene transfer have beneficial effects on EPC biology, relative myocardial vascularity, and diastolic function. These findings raise concerns over the external validity of studies evaluating myocardial biology and cardiac repair in normocholesterolemic animals.

## Introduction

Hypercholesterolemia and low high density lipoprotein (HDL) cholesterol contribute to coronary heart disease but little is known about their direct effects on myocardial function. Based on echocardiographic data, an early cardiomyopathy characterized by systolic and diastolic dysfunction was described in patients with primary hypercholesterolemia without evidence of coronary heart disease [1]. In patients with a first myocardial infarction, post-infarct left ventricular ejection fraction is adversely influenced by elevated non-HDL cholesterol levels and lower HDL cholesterol levels irrespective of the severity of coronary atherosclerosis [2], [3]. Low HDL and raised non-HDL cholesterol levels carried increased heart failure risk during follow-up in the Framingham study independent of any association with myocardial infarction [4].

Experimental data in rabbits support the hypothesis that hypercholesterolemia has direct effects on the myocardium. Hypercholesterolemia in rabbits induces electrical remodeling of the heart, characterized by prolongation of the action potential and of the heart rate corrected QT interval, increased repolarization dispersion, and vulnerability to ventricular fibrillation [5]. Using tissue Doppler imaging, systolic and diastolic dysfunction has been demonstrated in hypercholesterolemic rabbits [6]. Data on the effect of cholesterol on *in vivo* systolic and diastolic function are paralleled by observations in isolated cardiomyocytes showing a decrease in the maximum rate of cardiomyocyte shortening and the maximum rate of cardiomyocyte relaxation [7], [8]. Based on these observations, the term ‘cholesterol cardiomyopathy’ has been introduced [7]. However, these rabbit studies should be interpreted with caution. Indeed, plasma cholesterol levels in these studies were approximately 500 mg/dl [6] and 800 mg/dl [7], which is rarely observed in humans.

Communication between endothelial cells and cardiomyocytes regulates not only early cardiac development but also adult cardiomyocyte function [9]. Endothelial progenitor cells (EPCs) are part of the bone marrow-cardiac axis [10]. Paracrine interactions of these cells with resident endothelial cells and cardiomyocytes are a determinant of myocardial biology [11], [12]. In the current study, we evaluated the effect of lipid lowering gene transfer and HDL raising gene transfer on EPC number and EPC function, on relative myocardial vascularity, and on cardiac function in C57BL/6 low density lipoprotein receptor deficient (LDLr^−/−^) mice. Our hypothesis was that increased EPC number and EPC function after lipid lowering or HDL raising gene transfer may result in an enhanced relative vascularity in the myocardium and in an improved cardiac function.

## Materials and Methods

### Ethics Statement

All experimental procedures in animals were performed in accordance with protocols approved by the Institutional Animal Care and Research Advisory Committee of the Catholic University of Leuven.

### Animals

Female C57BL/6 LDLr^−/−^ mice, originally purchased from Jackson Laboratories (Bar Harbor, ME, USA), received a diet containing 0.2% (w/w) cholesterol and 10% (v/w) coconut oil *ad libitum* starting from the age of 12 weeks.

### Gene Transfer Vectors

The E1E3E4-deleted adenoviral vector AdLDLr [13] contains an 890 bp human *α_1_-antitrypsin* promoter and two copies of the 160 bp α_1_-*microglobulin* enhancer (constituting together the DC172 promoter [14]) upstream of the 5′ UTR of the human *apo A-I* gene, the full-length 2.6 kb *low density lipoprotein receptor* cDNA sequence, and 2 copies of the 774 bp human *hepatic control region-1*. Similarly, the E1E3E4-deleted adenoviral vector AdA-I [14] comprises the DC172 promoter upstream of the genomic human *apo A-I* sequence and 2 copies of the *hepatic control region-1*. The E1E3E4-deleted control vector Adnull [15] does not contain an expression cassette. Vector production was performed as described previously [15].

### In vivo Gene Transfer

Gene transfer in C57BL/6 LDLr^−/−^ mice was performed 3 weeks after start of the diet by tail vein injection of 5×10^10^ adenoviral particles of AdLDLr or AdA-I. Control mice were injected with saline or with 5×10^10^ adenoviral particles of the control vector Adnull [15]. Since no difference occurred between the Adnull and saline injected mice with regard to different end-points, data of both control groups were consistently pooled.

### Blood Sampling

Blood was obtained by puncture of the retro-orbital plexus. Anticoagulation was performed with 0.1 volume of 4% trisodium citrate and plasma was immediately isolated by centrifugation at 1100 g for 10 min and stored at −20°C.

### Plasma Lipid Analysis

Mouse lipoproteins were separated by density gradient ultracentrifugation in a swing-out rotor as described before [16]. Fractions were stored at −20°C until analysis. Total cholesterol in plasma and lipoprotein fractions was determined with commercially available enzymes (Roche Diagnostics, Basel, Switzerland). Precipath L (Roche Diagnostics) was used as a standard.

### HDL Isolation by Density Gradient Ultracentrifugation

Plasma HDL (1.063 g/ml<d<1.21 g/ml) was isolated from human plasma by density gradient ultracentrifugation in a swing-out rotor as described [16]. HDL was subsequently dialysed against PBS and concentrated. Protein concentration of HDL was determined using a bicinchoninic protein assay kit (Pierce Biotechnology Inc., Rockford, IL, U.S.A.).

### Murine Endothelial Progenitor Cell (EPC) Culture Assay

Spleen mononuclear cells were cultivated and EPCs were quantified as described before by Dimmeler *et al*. [17]. Spleen mononuclear cells were isolated 14 days after gene transfer by Ficoll-based centrifugation and seeded onto fibronectin (40 µg/ml)-coated 24-well plates (Sigma, Steinheim, Germany) at a density of 8×10^6^ cells/well in 0.5 ml EGM-2MV BulletKit medium (Cambrex, East Rutherford, NJ, U.S.A.) according to the instructions of the manufacturer. After 7 days of culture, medium was removed and adhered cells were stained for DiI-acLDL (Invitrogen, Carlsbad, CA, U.S.A.) (6.6 µg/ml) for 4 hours and then FITC-labeled isolectin (Invitrogen) (10 µg/ml) for 1 hour. The number of EPCs, identified as DiI-acLDL isolectin double positive cells, per microscopy field was quantified.

### EPC Migration Assay

EPC migration was studied by using modified Boyden chambers (Costar, Avon, France) as described [18]. After 7 days of culture, spleen EPCs were seeded in the upper chamber with a density of 2×10^4^ cells per well in 200 µl EGM-2MV medium. Five hundred µl EGM-2MV medium supplemented with HDL (100 µg/ml) or an equivalent amount of bovine serum albumin (Roche, Mannheim, Germany) was placed in the lower chamber. EPCs were allowed to migrate for 5 hours at 37°C. For quantification, cell nuclei were stained with 4′,6-diamidine-2-phenylidole dihydrochloride (DAPI; Invitrogen) and EPCs migrated into the lower chamber were counted manually in randomly selected microscopy fields. To investigate whether potentiation of EPC migration induced by HDL or by lipid lowering gene transfer depends on nitric oxide (NO), the NO synthase inhibitor N^G^-monomethyl-L-arginine (2 mM; L-NMA, Steinheim, Germany) was added to the lower chamber in selected experiments.

### EPC Adhesion Assay

The *in vitro* adherence of EPCs to fibronectin coated plates was performed as described [18]. In brief, after 7 days of culture, spleen EPCs were collected and 2×10^4^ cells per well were allowed to adhere onto fibronectin (40 µg/ml)-coated 96-well plates for 30 min in the presence 100 µg/ml of HDL or an equivalent amount of bovine serum albumin. Subsequently, the plates were vigorously washed with PBS and the number of adherent cells was counted under the microscope. To investigate whether potentiation of EPC adhesion by lipid lowering gene transfer depends on NO, the NO synthase inhibitor N^G^-monomethyl-L-arginine (2 mM; L-NMA) was added in selected experiments.

### EPC Survival Assay

Murine spleen mononuclear cells were seeded onto fibronectin-coated (40 µg/ml) 48-well plates at a density of 1.5×10^5^ cells/well containing 200 µl EGM-2MV BulletKit medium. After 7 days of culture, medium was removed, cells were washed with PBS and subsequently cultured for 24 hours in 200 µl EGM-2MV medium or medium without serum and growth factors either supplemented with HDL (100 µg/ml) or bovine serum albumin (100 µg/ml). Next, the medium was removed and cells were extensively washed. Adherent cells were fixed with a 4% paraformaldehyde solution and stained with FITC-labeled isolectin (Invitrogen) (10 µg/ml) for 1 hour. The number of positive cells per microscopy field was quantified in a blinded fashion.

### Bone Marrow EPC Isolation and Quantification

Bone marrow mononuclear cells were isolated by density gradient centrifugation using Histopaque-1077 (Sigma) as described [19]. Immediately following isolation, cells were plated onto fibronectin-coated (40 µg/ml) 24-well plates at a density of 4×10^6^ cells/well and cultured in EGM-2MV BulletKit medium (Cambrex). After 7 days of culture, the number of EPCs, identified as Dil-ac-LDL isolectin double positive cells, was quantified in randomly selected microscopy fields.

### Tissue Preparation for Histological Analysis

Hearts were harvested for histological analysis 6 weeks after gene transfer or saline injection. Mice were perfused via the abdominal aorta with phosphate-buffered saline (PBS) and hearts were arrested in diastole by CdCl (100 µl; 0.1 N), followed by perfusion fixation with 1% paraformaldehyde in PBS. After dissection, hearts were post-fixated overnight in a 1% paraformaldehyde solution, embedded in paraffin, and 6 µm thick cross-sections at 130 µm spaced intervals were made extending from the apex to the basal part of the left ventricle.

### Morphometric Analysis of the Myocardium

Laminin staining was performed with rabbit anti-mouse laminin antibodies (Sigma; 1/50). Cardiomyocyte cross-sectional area (µm^2^) was analyzed on laminin stained sections by measuring at least 200 randomly selected cardiomyocytes in the myocardium. Two mid-ventricular cross-sections were analyzed per mouse. Cardiomyocyte density was determined on the same laminin stained sections by counting the number of cross-sectioned round shaped cardiomyocytes per mm^2^ of LV myocardium. Relative vascularity of the myocardium was determined as [(capillary density (number/mm^2^)/cardiomyocyte density (number/mm^2^)/cardiomyocyte cross-sectional area (µm^2^)] [20] and was assessed on sections double stained for rat anti-mouse CD31 (BD Biosciences, Erembodegem, Belgium; 1/500) and rabbit anti-mouse laminin. Computer-assisted image analysis was performed using KS300 software (Zeiss, Zaventem, Belgium).

### In vivo Hemodynamic Measurements

Invasive hemodynamic measurements were performed 6 weeks after gene transfer or saline injection according to a protocol that has been described before [21]. Briefly, mice were anesthetized by intraperitoneal administration of 1.4 g/kg urethane (Sigma). Body temperature was maintained with a heating pad and monitored with a rectal probe. An incision in the right carotid artery was made with a 26-gauge needle between a distal and proximal non-occlusive ligation of the artery. A 1.1 French Millar pressure catheter (SPR-67/NR; Millar instruments, Houston, Texas, USA) was inserted and advanced to the left ventricle (LV). After stabilization of the catheter, heart rate, maximal systolic LV pressure, minimal diastolic LV pressure, the peak rate of isovolumetric LV contraction (dP/dt_max_), and the peak rate of isovolumetric LV relaxation (dP/dt_min_) were measured. The end-diastolic LV pressure was calculated manually from the pressure in function of time curves. The time constant of isovolumetric LV pressure fall (tau) was calculated using the method of Weiss *et al.* [22]. Arterial blood pressure measurements were obtained after withdrawal of the catheter from the LV to the ascending aorta. Data were registered with a Powerlab Bridge Amplifier and Chart Software (sampling rate 2000 Hz; Fysicon, Oss, the Netherlands).

### Real-time Quantitative Reverse Transcriptase Polymerase Chain Reaction Analysis

Six weeks after gene transfer or saline injection, hearts were dissected, briefly rinsed with saline buffer, snap-frozen, and stored at −80°C until use. RNA was extracted from the left ventricular myocardium using TRIzol reagent (Invitrogen, Carlsbad, CA, USA) and the Purelink™ RNA Mini Kit (Invitrogen). An on-column DNase treatment was performed using Purelink™ DNase (Invitrogen) according to the manufacturer’s protocol. Total RNA (1 µg) was reverse transcribed using the QuantiTect Reverse Transcription kit (Qiagen, Hamburg, Germany). Real-time quantitative reverse transcriptase-polymerase chain reaction (qRT-PCR) was performed on a 7500 FAST real-time PCR system (Applied Biosystems, Carlsbad, CA, USA) using the TaqMan Fast Universal PCR Master Mix (Applied Biosystems) and a premade mix containing primers and MGB probes (Taqman gene expression assay, Applied Biosystems; see Table S1 for details) to quantify *Atp2a2* and *Nos3* cDNA levels (n = 6 per group). The *glyceraldehyde 3-phosphate dehydrogenase (Gapdh)* housekeeping gene was used as endogenous control. Data analysis was performed using ΔΔCt-based fold-change calculations.

### Quantitative Western Blot Analysis

Left ventricular samples were homogenized in lysis buffer containing proteinase and phosphatase inhibitors. An equal amount of protein (20 µg) was loaded into a Novex 4–20% Tris-Glycine gel (Invitrogen™, Ghent, Belgium). Total Akt (Cell Signaling Technology, Leiden, the Netherlands), phosphorylated Akt-Ser 473 (Cell Signaling Technology), total endothelial nitric oxide synthase (eNOS) (BD Biosciences), phosphorylated eNOS-Ser 1177 (BD Biosciences), and GAPDH (Abcam, Cambridge, UK) were detected with corresponding specific antibodies. For the investigation of eNOS homodimer formation, non-boiled proteins were resolved by Novex 6% Tris-Glycine gel (Invitrogen™) at 4°C. Membranes were developed with chemiluminescene (ECL) system (GE Healthcare, Diegem, Belgium). Films were scanned and quantifications were performed with Image Lab 4.0 Software.

### Statistical Analysis

All data are expressed as means ± standard error of the means (SEM) with indication of sample size. Parameters between two groups were with an unpaired Student’s t-test using Instat3 (GraphPad Software, San Diego, CA, USA). When indicated, a logarithmic transformation, a square root transformation, or a non-parametric Mann-Whitney test was performed. Parameters between three groups were compared by one-way analysis of variance followed by Dunnett multiple comparisons test or Tukey multiple comparisons test. A two-sided p-value of less than 0.05 was considered statistically significant.

## Results

### LDLr Gene Transfer Lowers Plasma Lipoprotein Levels whereas Human Apo A-I Gene Transfer Selectively Increases HDL Cholesterol Levels

To induce pathophysiologically relevant levels of hypercholesterolemia, female C57BL/6 LDLr^−/−^ mice were fed a diet containing 0.2% (w/w) cholesterol and 10% (v/w) coconut oil. Three weeks after start of the diet, mice were treated with the LDLr expressing vector AdLDLr or the human apo A-I expressing vector AdA-I. Control mice were injected with the control vector Adnull or with saline buffer. Lipoprotein levels in control mice, AdLDLr mice, and AdA-I mice two weeks after saline injection or gene transfer are summarized in Table 1. Compared to control mice, plasma non-HDL cholesterol levels were 7.2-fold (p<0.01) lower in AdLDLr mice. This decrease corresponded to a 6.8-fold (p<0.01), 9.9-fold (p<0.01), and 5.3-fold (p<0.01) decline of plasma VLDL cholesterol, IDL cholesterol, and LDL cholesterol, respectively. AdA-I gene transfer resulted in a 1.8-fold (p<0.01) increase of HDL cholesterol levels in the absence of an alteration of non-HDL cholesterol levels (Table 1). Cholesterol levels remained stable in control, AdLDLr, and AdA-I mice until 6 weeks after saline injection or gene transfer, which corresponds to the end of the experiment.

**Table 1:** Total and non-HDL, VLDL, IDL, LDL, and HDL plasma cholesterol (mg/dl) at day 14 after saline injection or gene transfer with 5×10^10^ particles of Adnull (Controls), after gene transfer with 5×10^10^ particles of AdLDLr, and after gene transfer with 5×10^10^ particles of AdA-I in female C57BL/6 LDLr^−/−^ mice fed a diet containing 0.2% cholesterol, 10% coconut oil.

	Controls	AdLDLr	AdA-I
**Total**	353±10	81.7±4.5**	390±16*
**Non-HDL**	302±9	42.0±3.4**	298±11
**VLDL**	65.4±5.9	9.62±1.25**	69.2±3.5
**IDL**	138±4	13.9±1.1**	133±5
**LDL**	98.8±3.5	18.5±1.7**	96.0±2.6
**HDL**	51.0±1.9	39.7±1.6*	91.3±5.1**

Data are expressed as means ± SEM (n = 10 for each condition). Lipoproteins were isolated by ultracentrifugation.

* :p<0.05,

** :p<0.01 for comparison versus Controls.

### LDLr Gene Transfer Increases EPC Number and Improves EPC Function

**Figure 1:**
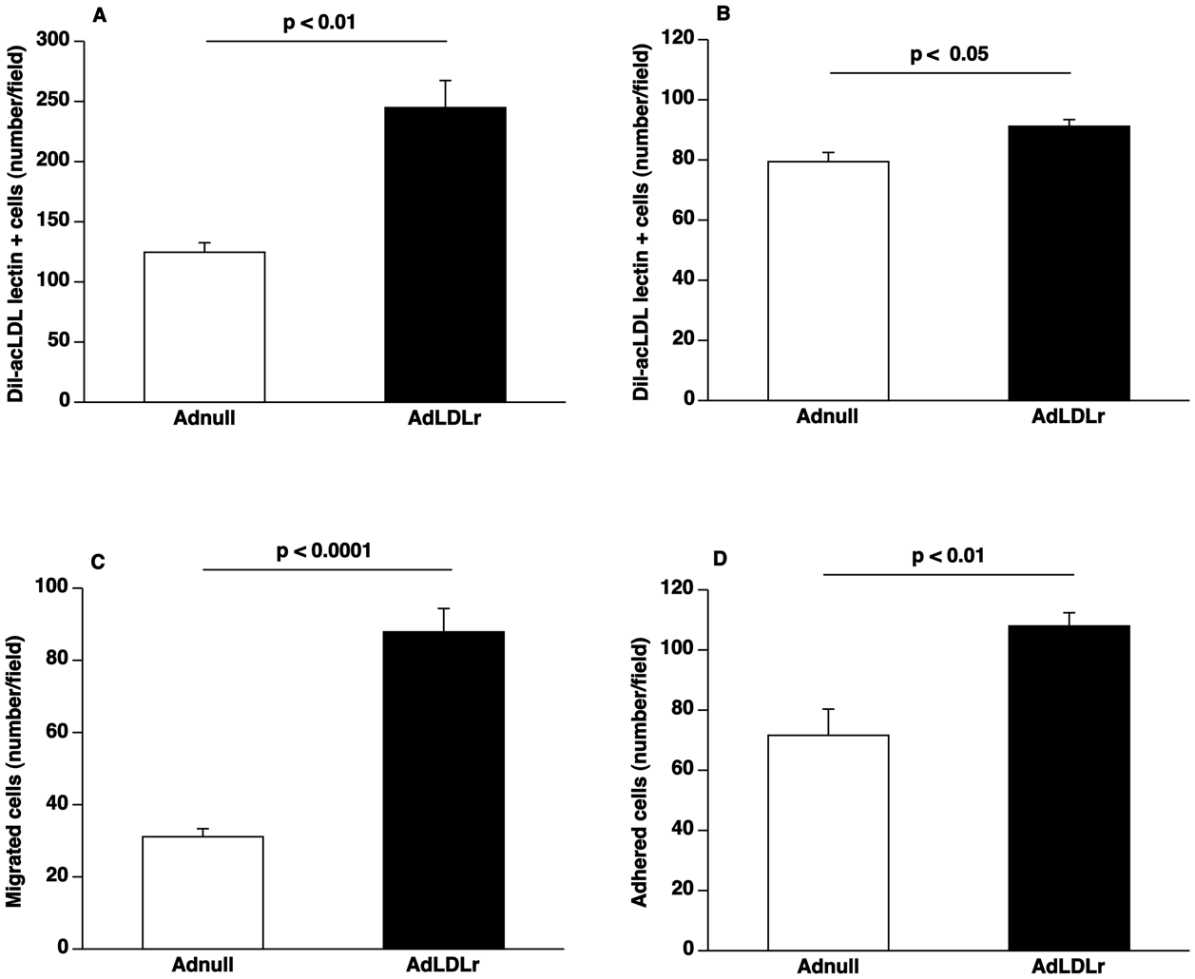
Lipid lowering gene transfer increases EPC number and enhances EPC function. (**A**) Bar graph showing the number of Dil-acLDL FITC-isolectin double positive cells after 7 days of *ex vivo* culture of spleen mononuclear cells isolated at day 14 after Adnull transfer or AdLDLr transfer in C57BL/6 LDLr^−/−^ mice (n = 5 for each group). (**B**) Bar graph illustrating the number of Dil-acLDL FITC-isolectin double positive cells after 7 days of *ex vivo* culture of bone marrow mononuclear cells isolated at day 14 after Adnull transfer or AdLDLr transfer in C57BL/6 LDLr ^−/−^ mice (n = 6 for each group). (**C**) Bar graph showing the number of migrated EPCs in modified Boyden chambers. After 7 days of culture, spleen EPCs isolated at day 14 after transfer from Adnull mice or AdLDLr treated C57BL/6 LDLr^−/−^ mice were seeded in the upper chamber. The number of migrated cells per microscopy field was quantified after 5 hours (n = 12 for each group). (**D**) Bar graph illustrating the number of EPCs adhered to fibronectin-coated plates. After 7 days of culture, spleen EPCs isolated at day 14 after transfer from Adnull injected mice (n = 6) or AdLDLr treated mice (n = 6) were allowed to adhere onto fibronectin-coated plates for 30 minutes. Following vigorously washing with PBS, the number of adherent cells was counted under the microscope. Data are expressed as means ± SEM.

To evaluate the effect of lipid lowering on the number of circulating EPCs, mononuclear cells were isolated from spleens of AdLDLr or Adnull treated C57BL/6 LDLr^−/−^ mice at day 14 after gene transfer. After 7 days of culture, EPC number was analyzed by quantification of Dil-acLDL FITC-isolectin double positive cells. The number of spleen EPCs was 2.0-fold higher (p<0.05) after AdLDLr transfer (n = 5) than after Adnull transfer (n = 5) (Figure 1A). There was a 15% (p<0.05) increase of EPCs in the bone marrow of AdLDLr mice (n = 6) compared to Adnull mice (n = 6) (Figure 1B). EPC number in the peripheral blood and in the bone marrow was not significantly different in Adnull injected mice compared to saline injected mice (data not shown). To analyse the effect of AdLDLr gene transfer on EPC function, EPC migration and EPC adhesion were quantified. The number of migrated EPCs isolated from AdLDLr treated mice (n = 12) was 2.8-fold (p<0.0001) higher compared to EPCs derived form Adnull treated mice (n = 12) (Figure 1C). Adherence to fibronectin-coated plates was 1.5-fold (p<0.01) higher for EPCs isolated from AdLDLr treated mice (n = 6) than for EPCs derived from Adnull injected mice (n = 6) (Figure 1D). Taken together, lipid lowering induces a marked increase of EPC number and significantly improves EPC function.

### Selective Increase of HDL Cholesterol Following Human Apo A-I Gene Transfer Increases EPC Number and Improves EPC Function

**Figure 2:**
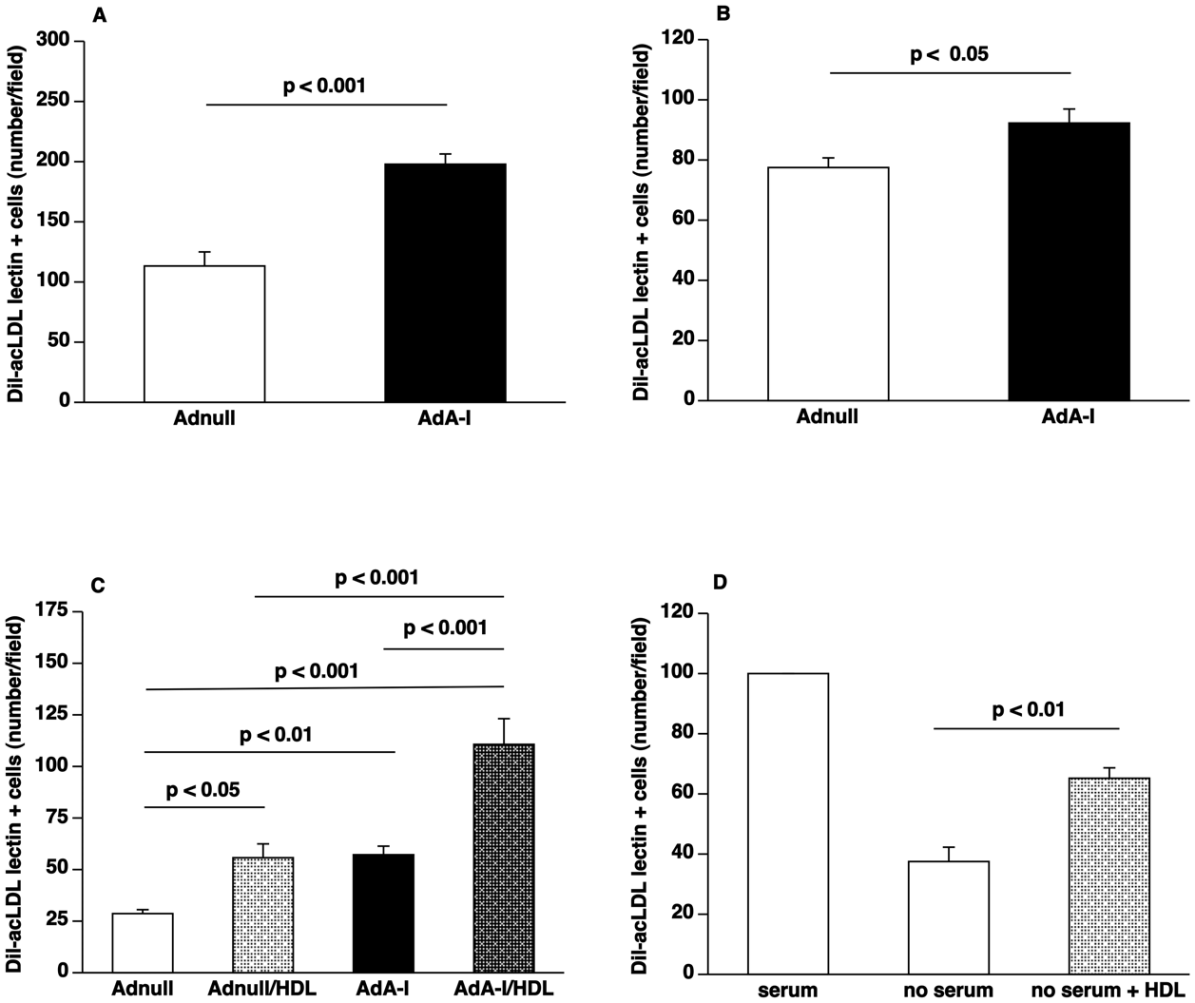
HDL raising gene transfer increases EPC number and enhances EPC function. (**A**) Bar graph showing the number of Dil-acLDL FITC-isolectin double positive cells after 7 days of *ex vivo* culture of spleen mononuclear cells isolated at day 14 after Adnull transfer or AdA-I transfer in C57BL/6 LDLr^−/−^ mice (n = 6 for each group). (**B**) Bar graph illustrating the number of Dil-acLDL FITC-isolectin double positive cells after 7 days of *ex vivo* culture of bone marrow mononuclear cells isolated at day 14 after Adnull transfer or AdA-I transfer in C57BL/6 LDLr^−/−^ mice (n = 10 for each group). (**C**) Bar graph showing the number of migrated EPCs in modified Boyden chambers. After 7 days of culture, spleen EPCs isolated from Adnull injected mice (n = 5) or AdA-I treated mice (n = 6) were seeded in the upper chamber. The lower chamber was supplemented with either HDL (100 µg/ml) or an equivalent amount of bovine serum albumin and the number of migrated cells per microscopy field was quantified after 5 hours. (**D**) Bar graph illustrating the number of surviving murine EPCs. After 7 days of culture, spleen EPCs isolated from C57BL/6 LDLr^−/−^ mice (n = 5) were cultured for 24 hours in EGM-2MV medium or medium without serum and growth factors either supplemented with HDL (100 µg/ml) or bovine serum albumin (100 µg/ml). The number of FITC-isolectin positive cells per microscopy field was quantified after 24 hours in a blinded fashion.

EPC number, defined as the number of Dil-acLDL FITC-isolectin double positive cells after 7 days of *ex vivo* cell culture, was 1.7-fold (p<0.001) higher in AdA-I treated mice (n = 6) compared to Adnull injected mice (n = 6) (Figure 2A). There was a 19% (p<0.05) increase of EPCs in the bone marrow of AdA-I mice (n = 10) compared to Adnull mice (n = 10) (Figure 2B).

To investigate the effect of human apo A-I transfer and HDL cholesterol on the function of EPCs, EPC migration and EPC adhesion were analysed *ex vivo*. The number of migrated EPCs isolated from AdA-I treated mice (n = 6) was 2.0-fold (p<0.01) higher compared to EPCs isolated from Adnull treated mice (n = 5) (Figure 2C). Addition of 100 µg/ml HDL to the lower chamber increased migration 1.9-fold (p<0.05) and 1.9-fold (p<0.001) for EPCs isolated from Adnull and AdA-I mice, respectively (Figure 2C). In the presence of HDL, the number of migrated EPCs was 2.0-fold (p<0.001) higher for cells isolated from AdA-I mice compared to cells isolated from Adnull mice (Figure 2C). Taken together, the intrinsic EPC function is higher in AdA-I treated mice and addition of HDL results in a proportional increase of EPC function *ex vivo*.

Figure 2D illustrates that under conditions of serum and growth factor deprivation HDL (100 µg/ml; n = 5) increased the percentage of surviving EPCs after 24 hours 1.7-fold (p<0.01) compared to controls without addition of HDL (100 µg/ml; n = 5). In summary, HDL significantly increases EPC number and improves EPC function.

### Enhanced EPC Function Induced by HDL and by LDLr Gene Transfer is Dependent on Nitric Oxide

**Figure 3:**
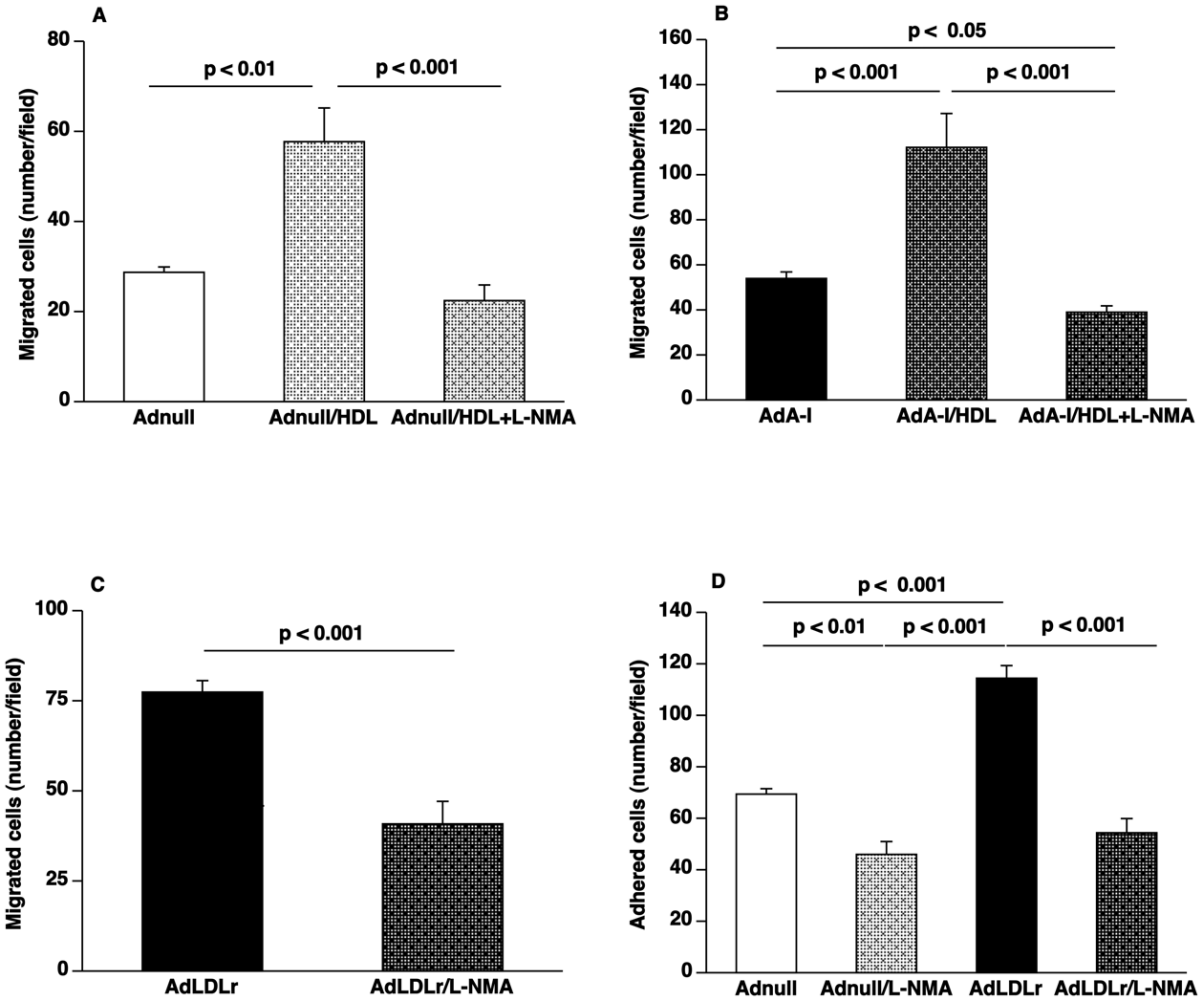
Potentiation of EPC function by HDL and by lipid lowering gene transfer is abrogated by the NO synthase inhibitor L-NMA. (**A**) Bar graph showing the number of migrated EPCs in modified Boyden chambers. After 7 days of culture, spleen EPCs isolated at day 14 after Adnull transfer (n = 6) were seeded in the upper chamber. The lower chamber was supplemented with either bovine serum albumin (100 µg/ml) or with HDL (100 µg/ml) or with HDL (100 µg/ml) in combination with 2 µM L-NMA. The number of migrated cells per microscopy field was quantified after 5 hours. (**B**) Bar graph showing the number of migrated EPCs in modified Boyden chambers. After 7 days of culture, spleen EPCs isolated at day 14 after AdA-I transfer (n = 6) were seeded in the upper chamber. The lower chamber was supplemented with either bovine serum albumin (100 µg/ml) or with HDL (100 µg/ml) or with HDL (100 µg/ml) in combination with 2 µM L-NMA. The number of migrated cells per microscopy field was quantified after 5 hours. (**C**) Bar graph showing the number of migrated EPCs in modified Boyden chambers. After 7 days of culture, spleen EPCs isolated at day 14 after AdLDLr gene transfer (n = 6) were seeded in the upper chamber. To inhibit NO synthase activity, the lower chamber was supplemented with L-NMA (2 µM) in selected experiments. (**D**) Bar graph illustrating the number of EPCs adhered to fibronectin-coated plates. After 7 days of culture, spleen EPCs isolated at day 14 after Adnull transfer (n = 6) or AdLDLr transfer (n = 6) were allowed to adhere onto fibronectin-coated plates for 30 minutes. To inhibit NO synthase activity, the lower chamber was supplemented with L-NMA (2 µM) in selected experiments. Following vigorously washing with PBS, the number of adherent cells was counted under the microscope. Data are expressed as means ± SEM.

Figure 3 illustrates the results of EPC experiments designed to evaluate to which extent the observed beneficial effects of HDL and of lipid lowering gene transfer on EPC function are mediated via nitric oxide (NO). Potentiation of *ex vivo* migration of EPCs isolated from Adnull mice (n = 6) or from AdA-I mice (n = 6) by addition of 100 µg/ml HDL was confirmed in independent experiments (Figure 3A and Figure 3B). This HDL induced EPC migration was completely abrogated in the presence of the NO synthase inhibitor N^G^-monomethyl-L-arginine (2 mM; L-NMA). Potentiation of EPC migration by HDL was decreased by 61% (p<0.001) (Figure 3A) and by 65% (p<0.001) (Figure 3B) for EPCs isolated from Adnull mice and AdA-I mice, respectively, by addition of L-NMA. Migration of EPCs isolated from AdLDLr mice was reduced by 47% (p<0.001) in the presence of L-NMA (Figure 3C). The effect of L-NMA on EPC adhesion was evaluated for EPCs isolated from Adnull mice and AdLDLr mice (Figure 3D). In the presence of L-NMA, EPC adhesion was reduced by 34% (p<0.01) and by 52% (p<0.001) for EPCs isolated from Adnull and AdLDLr mice, respectively. EPC adhesion was not significantly different between EPCs isolated from Adnull mice and AdLDLr mice in the presence of L-NMA. Taken together, these results suggest that the ‘priming’ effect observed in EPCs isolated from AdLDLr mice is dependent on enhanced NO production.

### Both Lipid Lowering and HDL Raising Gene Transfer Increase Capillary Density and Relative Vascularity in the Myocardium

Coronary atherosclerosis was completely absent in all groups (data not shown). Morphometric analysis of the myocardium was performed in C57BL/6 LDLr^−/−^ mice 6 weeks after saline injection or gene transfer with Adnull (controls), after gene transfer with AdLDLr, and after gene transfer with AdA-I. Data are summarized in Table 2. Capillary density and cardiomyocyte density were 28% (p<0.01) and 20% (p<0.05) higher in AdLDLr mice compared to control mice. The relative vascularity of the myocardium, defined as capillary density (number/mm^2^)/cardiomyocyte density (number/mm^2^)/cardiomyocyte cross-sectional area (µm^2^), increased by 22% (p<0.05) in AdLDLr mice compared to control mice. In AdA-I mice, capillary density was 24% (p<0.05) higher than in controls and this was paralleled by 24% (p<0.05) increase of relative vascularity (Table 2). Taken together, both non-HDL and HDL lipoproteins significantly affect relative myocardial vascularity in mice.

**Table 2:** Morphometric analysis of the myocardium in C57BL/6 LDLr^−/−^ mice 6 weeks after saline injection or gene transfer with Adnull (controls), gene transfer with AdLDLr, and gene transfer with AdA-I.

	Controls n = 13	AdLDLr n = 14	AdA-I n = 10
Cardiomyocyte cross-sectional area (µm^2^)	219±18	184±10	188±8
Cardiomyocyte density (number/mm^2^)	4490±300	5410±230*	4900±180
Capillary density (number/mm^2^)	5820±360	7460±330**	7190±410*
Capillary density/cardiomyocyte density	1.29±0.05	1.38±0.04	1.46±0.05*
Relative vascularity (µm^−2^)	0.00639±0.00045	0.00782±0.00043*	0.00794±0.00046*

Data are expressed as means ± SEM.

* :p<0.05,

** :p<0.01 for comparison versus Controls.

### Both Lipid Lowering and HDL Raising Gene Transfer Improve Diastolic Function in Mice

Hemodynamic parameters were analyzed in C57BL/6 LDLr^−/−^ mice 6 weeks after saline injection or gene transfer with Adnull (controls), after gene transfer with AdLDLr, and after gene transfer with AdA-I. Data are summarized in Table 3. The peak rate of isovolumetric relaxation (dP/dt min) was increased by 12% (p<0.05) and by 16% (p<0.05) in AdLDLr mice and AdA-I mice, respectively, compared to control mice (Table 3). The time constant of isovolumetric relaxation was 14% (p<0.05) lower in AdLDLr mice and reduced by 13% (p = NS) in AdA-I mice compared to control mice (Table 3). Taken together, diastolic function in C57BL/6 LDLr^−/−^ mice is significantly improved by lipid lowering or by a selective increase of HDL.

**Table 3:** Hemodynamic parameters in C57BL/6 LDLr^−/−^ mice 6 weeks after saline injection or gene transfer with Adnull (controls), gene transfer with AdLDLr, and gene transfer with AdA-I.

	Controlsn = 13	AdLDLrn = 14	AdAI n = 10
**LEFT VENTRICLE**			
Mean pressure (mm Hg)	38.1±0.9	38.1±1.1	39.7±1.6
Peak systolic pressure(mm Hg)	100±1	99.4±2.2	103±2
End-diastolic pressure(mm Hg)	1.79±0.63	0.844±0.506	1.59±0.74
Peak dP/dt max(mm Hg/ms)	9.96±0.45	10.2±0.3	10.6±0.7
Peak dP/dt min(mm Hg/ms)	−7.96±0.30	−8.93±0.14*	−9.21±0.44*
Tau (ms)	5.16±0.22	4.54±0.12*	4.6±0.21
Heart rate (bpm)	621±13	606±13	614±12
**AORTA**			
Mean pressure (mm Hg)	81.2±1.8	81.4±3.2	82.1±2.4
Peak systolic pressure(mm Hg)	99.6±1.9	99.6±3.0	102±2
Peak diastolic pressure(mm Hg)	63.9±2.3	63.7±3.8	64.6±2.8

Data are expressed as means ± SEM.

* :p<0.05 for comparison versus Controls.

### No Evidence for Enhanced Endothelial Nitric Oxide Synthase Activity in the Myocardium Following Lipid Lowering and HDL Raising Gene Transfer

*Endothelial NO synthase* (*eNOS*) mRNA levels in the left ventricular myocardial tissue quantified by real-time qRT-PCR were not significantly altered after AdLDLr or AdA-I transfer (Table 4). Akt can directly phosphorylate eNOS and activate the enzyme, leading to enhanced NO production. Phosphorylation of Akt (protein kinase B) and phosphorylation of eNOS in myocardial tissue of control mice, AdLDLr mice, and AdA-I mice was analyzed by quantitative Western blot (Table 5). No significant alteration of phosphorylation of Akt and eNOS was observed in myocardial tissue of AdLDLr mice and AdA-I mice compared to control mice. In addition, eNOS dimer and eNOS monomer levels were quantified. No significant differences of the eNOS dimer/monomer ratio occurred in AdLDLr mice and AdA-I mice (Table 5). Taken together, these data suggest that no significant augmentation of NO production occurs in the myocardium of AdLDLr mice and AdA-I mice.

**Table 4:** Analysis of mRNA expression levels of eNOS and SERCA2 in the left ventricular myocardium 6 weeks after gene transfer or saline injection.

	Controls	AdLDLr	AdA-I
eNOS	1.00±0.07	1.03±0.19	0.885±0.084
SERCA2	1.00±0.15	1.07±0.12	0.935±0.045

Gene expression data analysis was performed using ΔΔCt-based fold change-calculations. mRNA expression levels were first normalized to the GAPDH expression levels and data represent values relative to controls. Data are expressed as means ± SEM (n = 6 for each condition).

**Table 5:** Protein expression in the left ventricular myocardium evaluated by quantitative Western blot 6 weeks after gene transfer or saline injection.

	Controls	AdLDLr	AdA-I
Akt	1.17±0.34	0.918±0.067	0.984±0.151
Phospho-Akt	0.208±0.061	0.0154±0.037	0.183±0.039
Phospho-Akt/Akt (%)	17.7±5.2	17.1±4.0	21.5±6.0
eNOS	0.941±0.113	0.859±0.086	1.04±0.12
Phospho-eNOS	0.206±0.060	0.160±0.036	0.189±0.039
Phospho-eNOS/eNOS (%)	23.3±7.3	19.3±4.6	18.1±2.5
eNOS dimer/monomer ratio	0.695±0.150	0.428±0.118	0.622±0.115

The Akt, phospho-Akt, eNOS, and phospho-eNOS signals were normalized to the GAPDH signal. Data are expressed as means ± SEM (n = 6 for each condition).

Quantification of the *sarco-endoplasmic reticulum Ca^2+−^ATPase-2* (*Serca2*) mRNA in the left ventricular myocardial tissue by real-time qRT-PCR demonstrated no significant alterations after AdLDLr or AdA-I transfer (Table 4).

## Discussion

The main findings of the current study are that 1) both lipid lowering and HDL raising gene transfer markedly increase EPC number and enhance EPC function in C57BL/6 LDLr^−/−^ mice; 2) capillary density and relative vascularity in the myocardium are increased by lowering of non-HDL lipoproteins and raising of HDL lipoproteins; and 3) diastolic cardiac function is significantly improved following lipid lowering and HDL augmenting gene transfer.

In view of the importance of the bone marrow-cardiac axis, we first showed that both lipid lowering and HDL raising gene transfer increase EPC number and enhance EPC function in C57BL/6 LDLr^−/−^ mice fed a diet containing 0.2% cholesterol and 10% coconut oil. Plasma cholesterol levels in this model of hyperlipidemia were approximately 350 mg/dl, which is close to values observed in humans with severe hypercholesterolemia. The effect of human apo A-I gene transfer on EPCs in C57BL/6 LDLr^−/−^ mice is in line with prior studies in C57BL/6 apo E^−/−^ mice [18], [23]. Increased EPC number and improved EPC function may play an important role in the enhanced relative vascularity in the myocardium that was observed following LDLr and human apo A-I gene transfer in the current study. In addition, the relevance of these effects on EPCs should be seen in light of the stem/progenitor cell therapy field. Experimental animal studies have demonstrated that bone marrow-derived stem cells and EPCs may improve cardiac function after myocardial infarction [24]. However, the effects of stem/progenitor cell administration on cardiac function in the clinical setting have not met expectations. An important source of the discrepancy between the clinical setting and experimental animal studies may be that the latter studies were performed under conditions of normocholesterolemia. In the current study, we show that the lipoprotein profile has marked effects both on EPC biology and on cardiac structure. The compromised function of infused EPCs in patients with hypercholesterolemia and/or low HDL cholesterol might abrogate the beneficial effects that were observed in animal models.

The increased relative vascularity following LDLr and human apo A-I gene transfer may play an important role in cardiac remodeling induced by an increased load. Microvascular rarefaction and compromised myocardial perfusion are typically observed under conditions of structural remodeling of the myocardium under conditions of overload [25]. Physiological hypertrophy requires coordinated tissue growth and angiogenesis [26]. In pathological hypertrophy, mismatch between cardiomyocyte size and vascularity may induce myocardial hypoxia and cardiomyocyte death, which may accelerate progression to congestive heart failure [27]. Therefore, hypercholesterolemia is expected to speed up heart failure development under conditions of increased load, which was indeed observed in a model of post-myocardial infarction remodeling [21]. Furthermore, oxygen extraction of the myocardium at rest is approximately 75%. The limited extraction reserve of the heart implies that coronary blood flow must increase to match increased myocardial oxygen consumption during exercise [28]. We speculate that the decreased relative vascularity may contribute to coronary microvascular dysfunction [29] under conditions of hypercholesterolemia. In other words, irrespective of epicardial atherosclerotic lesions and endothelial dysfunction, hypercholesterolemia induces a diminished coronary flow reserve via a decreased relative vascularity index. The decreased coronary flow reserve will reduce the maximal amount of work that can be performed by the heart per unit of time.

Endothelial-cardiomyocyte communication involves neuregulin, vascular endothelial growth factor, angiopoietin, nitric oxide, angiotensin II, and other autocrine and paracrine factors [30]. Endothelial cells in the heart have therefore been proposed to play an obligatory role in regulating and maintaining cardiac function, in particular, at the endocardium and in the myocardial capillaries where endothelial cells directly interact with adjacent cardiomyocytes [30]. Release of nitric oxide catalyzed by eNOS in the coronary endothelium exerts paracrine effects on cardiomyocytes, predominantly affecting the timing of relaxation [31]. Our initial hypothesis was that AdLDLr and AdA-I transfer would result in enhanced NO production and increased NO bioavailability in the myocardium. Hypercholesterolemia may decrease NO production by promoting the interaction of caveolin and eNOS [32] and by oxidized LDL/CD36-induced redistribution of eNOS from caveolae leading to the inability to efficiently activate eNOS [33]. In contrast, HDL maintains the lipid environment in caveolae, thereby promoting the retention and function of eNOS in this domain [34]. HDL may also cause direct activation of eNOS via scavenger receptor type BI (SR-BI)-induced kinase signaling [34], [35]. However, our data suggest that no significant augmentation of NO production occurred in the myocardium of AdLDLr mice or AdA-I mice. We did not observe increased eNOS phosphorylation or an alteration of the eNOS dimer/monomer ratio following LDLr or HDL raising gene transfer. The absence of increased NO production and of increased NO bioavailability in LDLr mice is consistent with *ex vivo* aortic vasomotor experiments in hypercholesterolemic C57BL/6 LDLr^−/−^ mice [36] and C57BL/6 apo E^−/−^ mice [37] of similar age. In these studies [36], [37], normal acetylcholine-induced vasodilatation was observed in hypercholesterolemic mice compared to C57BL/6 mice, indicating normal NO bioavailability and normal endothelial function. Taken together, whereas enhanced eNOS activity plays a role in the beneficial effects of lipid lowering and HDL raising gene transfer on EPC function, the effect of these gene transfer interventions on cardiac function appears to be unrelated to eNOS activity and NO bioavailability in the myocardium. This suggests that improved cardiac function is related to NO independent endothelial-cardiomyocyte interactions or to direct effects of the interventions on cardiomyocytes.

Alterations of the cell membrane composition may significantly contribute to the observed effects of lipid lowering gene transfer on diastolic function. Myocardial free cholesterol levels are significantly elevated in hypercholesterolemic C57BL/6 LDLr^−/−^ mice compared to C57BL/6 mice [16] and similar observations have been performed in hypercholesterolemic rabbits [6], [7]. Lipid lowering gene transfer in C57BL/6 LDLr^−/−^ mice restored myocardial free cholesterol levels to values of wild-type C57BL/6 mice [16]. Cholesterol is a key structural component of membranes, particularly the plasma membrane. Structural membrane alterations induced by an increased membrane cholesterol content changes the physical properties of the membrane [38] and may affect the function of ion channels [39], [40] and pumps [7], [40]. Cholesterol-induced suppression of channel activity has e.g. been demonstrated for inwardly rectifying K^+^ (Kir) channels [41], [42], [43], Ca^2+^-sensitive K^+^ channels [44], N-type Ca^2+^ channels [45], and volume-regulated anion channels [46] in different types of cells. SERCA2 protein levels in the myocardium were lower and SERCA-mediated Ca^2+^ uptake into sarcoplasmic reticulum vesicles was impaired in hypercholesterolemic rabbits [7]. However, we did not do observe an alteration of *Serca2* mRNA levels after LDLr gene transfer.

Recently, it was demonstrated that reconstituted HDL shortened repolarization of isolated rabbit cardiomyocytes [47]. Moreover, infusion of reconstituted HDL shortened the heart-rate corrected QT interval on surface electrocardiograms in humans [47]. Abnormal ventricular repolarization in patients with long QT syndrome coincides with impaired diastolic function in a number of these patients [48]. Therefore, the effects on HDL on the action potential and surface electrocardiogram [47] appear to be concordant with the effects of raised HDL on diastolic function.

In conclusion, the current experimental intervention study shows that both lipid lowering and HDL raising gene transfer have beneficial effects on EPC biology, relative vascularity in the myocardium, and on diastolic function. The profound effects of hypercholesterolemia on these parameters raises concerns over the clinical relevance of studies evaluating myocardial biology and stem/progenitor cell therapy in normocholesterolemic animals.

## Supporting Information

Table S1 **Overview of Taqman gene expression assays used for quantitative reverse transcriptase polymerase chain reaction.**
(DOCX)
