# Supplementary material for: Lipid Lowering and HDL Raising Gene Transfer Increase Endothelial Progenitor Cells, Enhance Myocardial Vascularity, and Improve Diastolic Function
Source: PLoS One. 2012 Oct 4;7(10):e46849. doi: 10.1371/journal.pone.0046849 (PMC3464236; doi:10.1371/journal.pone.0046849)
Supplement: Table S1 — Overview of Taqman gene expression assays used for quantitative reverse transcriptase polymerase chain reaction. (DOCX) [file pone.0046849.s001.docx]

**Table S1.** Overview of Taqman gene expression assays used for quantitative reverse transcriptase polymerase chain reaction.

| **Gene symbol** | **Gene name** | **Gene aliases** | **GenBank accession #** | **Assay ID** | **Amplicon length** |
| --- | --- | --- | --- | --- | --- |
| *Atp2a2* | ATPase,Ca^2+^transportin, cardiac muscle, slow twitch 2 | SERCA2 | [NM_009722.3](http://www.ncbi.nlm.nih.gov/entrez/viewer.fcgi?val=NM_007527.3) | Mm01201431_m1 | 90 |
| *Gapdh* | glyceraldehyde-3-phosphate dehydrogenase | Gapd | [NM_008084.2](http://www.ncbi.nlm.nih.gov/nuccore/NM_008084.2) | Mm99999915_g1 | 107 |
| *Nos3* | Nitric oxide synthase 3 | eNOS | [NM_008713.4](http://www.ncbi.nlm.nih.gov/entrez/viewer.fcgi?val=NM_007742.3) | Mm00435217_m1 | 71 |
